# Awareness, Knowledge, and Uptake of the Human Papillomavirus (HPV) Vaccine Among Female Adolescents in the Rural Sonipat District of Haryana, India

**DOI:** 10.7759/cureus.96340

**Published:** 2025-11-07

**Authors:** Gayatri Mohanty, Anita Punia, SK Jha, Sanjeet Singh, Ilakkiya P, Sumit Goyat

**Affiliations:** 1 Community Medicine, Bhagat Phool Singh Government Medical College for Women, Khanpur Kalan, IND

**Keywords:** adolescents, awareness, hpv, hpv vaccine, knowledge

## Abstract

Background: Cervical cancer develops in the cells of the cervix, the lower part of the uterus that connects to the vagina. It is primarily caused by persistent infection with high-risk types of the human papillomavirus (HPV), a common sexually transmitted virus. Raising awareness about its symptoms, risk factors, and preventive measures can save lives, particularly in low-resource settings where the disease burden is higher. Our study aims to assess the awareness, knowledge, and uptake of the HPV vaccine among female adolescents, as well as its association with sociodemographic characteristics.

Methodology: A school-based analytical cross-sectional study conducted in India included 540 female adolescents. The collected data included sociodemographic information, knowledge of cervical cancer, HPV infection, HPV vaccination, and uptake of the HPV vaccine.

Results: The study's findings indicated that more than 80% of the participants had inadequate knowledge regarding cervical cancer and HPV infection, but nearly 60% of the participants had adequate knowledge about HPV vaccination. There was a statistically significant association of knowledge of cervical cancer with the number of siblings (p=0.022), knowledge of HPV infection with class (p=0.001), and HPV vaccine uptake.

Conclusion: Given the low uptake and knowledge levels, there is a critical need for comprehensive awareness campaigns and educational interventions targeting HPV and cervical cancer prevention. Further studies are needed to explore the factors influencing vaccine uptake and to evaluate the effectiveness of targeted educational programs to increase vaccine coverage among adolescents.

## Introduction

Cervical cancer develops in the cells of the cervix, the lower part of the uterus that connects to the vagina. It is primarily caused by persistent infection with high-risk types of the human papillomavirus (HPV), a common sexually transmitted virus. Raising awareness about its symptoms, risk factors, and preventive measures can save lives, particularly in low-resource settings where the disease burden is higher. According to the World Health Organization (WHO), cervical cancer is the fourth most common cancer among women globally, with an estimated 604,000 new cases and 342,000 deaths in 2020. The burden of cervical cancer disproportionately affects low- and middle-income countries (LMICs), which account for nearly 90% of cases. WHO has set a global strategy to eliminate cervical cancer as a public health problem, aiming to achieve 90% HPV vaccination coverage, 70% screening coverage, and 90% treatment for cervical pre-cancer and cancer by 2030 [1].

Over the past two decades, cervical cancer incidence and mortality have shown a declining trend in high-income countries, largely due to effective screening programs and widespread HPV vaccination [2]. India bears one of the highest burdens of cervical cancer globally, with approximately 662,301 new cases and 348,874 deaths annually, according to the Global Cancer Observatory (GLOBOCAN) 2022 data [3]. Cervical cancer ranks as the second most common cancer among Indian women aged 15-44 years. The age-standardized incidence rate in India is 18 per 100,000 women, significantly higher than the global average of 13.3 per 100,000 women, which accounts for approximately 17.7% of all new cancer cases in females according to the GLOBOCAN 2022 data. Despite advancements in healthcare, significant gaps in awareness, early detection, and vaccination remain prevalent across the country. Socioeconomic disparities further exacerbate the issue, with rural areas exhibiting higher disease burdens due to limited healthcare infrastructure and cultural barriers. Cultural barriers such as stigma and taboo significantly hinder open discussions on sexual and reproductive health. In many settings, topics related to reproductive organs or gynecological symptoms are considered inappropriate or embarrassing to talk about. Factors like modesty concerns, preference for female healthcare providers, patriarchal family structures where men make health-related decisions, reliance on traditional or supernatural explanations for illness, and a generally low perceived risk of disease further contribute to reduced health-seeking behavior and poor awareness among women.

In Haryana, cervical cancer contributes significantly to the cancer burden, though state-specific data remains less comprehensive than national figures. According to hospital-based cancer registries, cervical cancer is among the top three cancers affecting women in the state. In Sonipat district, anecdotal evidence and preliminary studies suggest low awareness levels and limited vaccine uptake. A 2021 survey conducted in select districts of Haryana indicated that less than 15% of women had undergone cervical cancer screening and HPV vaccination coverage was below 5% [4]. Awareness about cervical cancer and HPV infection varies widely across countries. In India, studies indicate that a significant proportion of women are unaware of cervical cancer and its association with HPV [5]. A study done in 2022, at Delhi and Rohtak (North India), found that only 10.9% of women had heard about the HPV vaccine. This provides additional evidence of the extremely low awareness of the vaccine in rural/non-metropolitan areas of India [6].

WHO recommends the HPV vaccine for girls aged 9-14 years before the onset of sexual activity [7]. In India, the vaccine was introduced in select states through pilot programs in 2008, but nationwide rollout has faced challenges due to cost, logistical barriers, and sociocultural resistance. As of 2023, only a few states have achieved moderate vaccination coverage, with significant disparities between urban and rural areas [8].

WHO's global strategy to eliminate cervical cancer aims to vaccinate 90% of girls by the age of 15 by 2030 [9]. Studies reveal low awareness and knowledge about the HPV vaccine among the Indian population [10]. In India, misconceptions and a lack of knowledge about HPV and its role in cervical cancer persist across both rural and urban settings. In rural areas, perceived susceptibility is often low due to limited education and awareness campaigns [5]. Conversely, urban populations may have a slightly higher perceived risk due to greater access to healthcare information and media. In India, cervical cancer remains the second most common cancer among women [11]. Among female adolescents, awareness levels are particularly low, with many unaware of the vaccine's existence and benefits. Our study aims to assess the awareness, knowledge, and uptake of the HPV vaccine among female adolescents and its association with sociodemographic characteristics.

## Materials and methods

Study design, setting, and population

This school-based analytical cross-sectional study was conducted between December 2023 and May 2024 in the field practice areas of rural health centers associated with Bhagat Phool Singh Government Medical College for Women, one of the government medical institutes in the Sonepat district of Haryana, India. The district is divided into eight educational blocks; out of them, one block falls under the field practice area of rural health centers, which is the Sonipat block. A total of 25 senior secondary schools were in the Sonipat block, according to the Haryana Education Department online portal. Five schools from all the senior secondary schools of the selected block were selected by the lottery method. The target population comprised adolescents, aged 10-19 years, studying in these schools. Inclusion criteria were all female students in 11th and 12th standards of senior secondary schools who were present on the day of data collection and willing to participate in the study. Those who could not understand the information, instructions, or questions related to a study or process were deliberately left out.

Sample size

The sample size for the quantitative study was determined by using a single population proportion formula, considering an 18% prevalence (p) estimate of HPV vaccine awareness [6], a 4% absolute error (d), and a 95% confidence level. Finally, a sample size of 532 adolescents was determined. A simple random sampling technique was adopted to select the students among those who were present on the day of data collection. 

Data collection and study instruments

Data collection was conducted through in-person interviews using a pre-tested, semi-structured questionnaire that covered aspects of sociodemographic characteristics and the knowledge of study participants about cervical cancer, HPV infection, and HPV vaccination (Appendix A and Appendix B).

Cervical Cancer

This part was assessed based on five questions. Each correct response was awarded 1 mark, while incorrect responses received 0 marks. Consequently, the maximum achievable score for this component was 9, yielding a potential score range of 0-9, with adequate knowledge defined as a score of ≥60% and inadequate knowledge defined as a score of <60%.

HPV Infection

This part was assessed based on four questions related to HPV infection, regardless of whether they were acquainted with HPV infection beforehand. For each correct response, 1 mark was given, and 0 was given for every incorrect response. The score ranges from 0 to 11, with adequate knowledge defined as a score of ≥60% and inadequate knowledge defined as a score of <60%.

HPV Vaccination

This part was assessed based on four questions asked to them, only when they had heard of this vaccine before. For each correct response, 1 mark was given, and 0 was given for every incorrect response. The score ranges from 1 to 6, with adequate knowledge defined as a score of ≥60% and inadequate knowledge defined as a score of <60%.

Statistical analysis

The quantitative data were checked for completeness and consistency of the information obtained from the respondent and entered into an Excel sheet (Microsoft Corporation, Redmond, Washington, United States). The data were checked for missed values and outliers. Descriptive analysis (like frequencies, tables, percentages, means, and standard deviation) was done to describe variables. Next, binary logistic regression was performed, and variables with a p-value of ≤0.25 were transported to binary logistic regression to identify significant factors associated with HPV vaccine awareness and uptake. Finally, variables with a p-value of ≤0.05 in the binary logistic regression model were taken as statistically significant, and the adjusted odds ratio with its 95% confidence interval was considered to see the association by using the R software version 4.3.2 (R Foundation for Statistical Computing, Vienna, Austria).

Ethical approval

Ethical approval was granted by the Institutional Ethics Committee of Bhagat Phool Singh Government Medical College for Women (approval number: BPSGMCW/RC919/IEC/23).

## Results

Out of 540 participants, nearly half (265) had heard about cervical cancer before, and among these 265 participants, the majority agreed that cervical cancer is a common female cancer. Seventy-one students knew that the cervix is the organ that is affected by this disease, and more than two-thirds of the students knew that the sexual route is the mode of transmission. Out of 265 students who gave multiple responses to risk factors for cervical cancer, the highest vote was given to "human papillomavirus infection" for being the risk factor given by more than half of the students, followed by "early age onset of sexual activity" by more than one-third, while 46 students didn't know any of the risk factors mentioned above. The respondents gave multiple responses on the question of recognizing signs and symptoms of cervical cancer, of which half of the vote was given to "abnormal vaginal bleeding", followed by "dyspareunia" (15.1%) and "vaginal discharge" (12.5%). A significant portion of the respondents did not know the signs and symptoms of cervical cancer, accounting for 32.1% (Table 1).

**Table 1:** Distribution of study participants according to knowledge of cervical cancer (n=540) *Multiple responses

Variables	Frequency (%)
Heard of cervical cancer before (n=540)	
Yes	265 (48.9)
No	275 (51.1)
Cervical cancer is a common female cancer (n=265)	
Yes	205 (77.4)
No	30 (11.3)
I don't know	30 (11.3)
Organs affected by cervical cancer	
Uterus	140 (52.8)
Breasts	25 (9.4)
I don't know	29 (10.9)
Others (specifying the cervix)	71 (26.9)
Mode of acquisition of cervical cancer	
Sexual transmission	187 (70.8)
History of cancer in the family	20 (7.2)
I don't know	58 (22)
What do you think is/are the risk factor(s) for cervical cancer?*	
Human papillomavirus infection	142 (53.6)
Promiscuity	34 (12.8)
Early age onset of sexual activity	98 (37)
Tobacco smoking	35 (13.2)
History of sexually transmitted infections	75 (28.3)
I don't know	46 (17.4)
When do you recognize the signs and symptoms of cervical cancer? When there is:*	
Abnormal vaginal bleeding	131 (49.4)
Vaginal discharge	33 (12.5)
Dyspareunia	40 (15.1)
I don't know	85 (32.1)

Among 540 students, two-fifths had heard of HPV causing sexually transmitted infections. Almost one-third of the respondents answered that both men and women can contract HPV infection. In multiple responses from 540 respondents regarding the diseases caused by HPV infection, nearly half of the participants voted cervical cancer, followed by penile cancer at 23.9%. Almost half of the respondents didn't know the answer to this question, which was asked. More than half of the participants did not know any answers to ways of preventing HPV diseases. The rest of the participants voted for "vaccination" (38.1%), the number one way for prevention, followed by more than one-third answered "using condoms". Each respondent gave multiple responses to risk factors for HPV infection, and it was found that more than half of them didn't know the answer. The rest who knew gave the highest vote to "high frequency of sex partner exchange" (34.8%) as the major risk factor (Table 2).

**Table 2:** Distribution of study participants according to knowledge of HPV infection (n=540) *Multiple responses HPV: human papillomavirus

Variables	Frequency (%)
HPV is a virus that causes sexually transmitted infections. Have you ever heard of it?	
Yes	227 (42)
No	313 (58)
Who can contract HPV infection?	
Only men	0 (0)
Only women	62 (11.5)
Men and women	204 (37.8)
I don't know	274 (50.7)
Which of these diseases are caused by HPV infection?*	
Cervical cancer	240 (44.5)
Penile cancer	129 (23.9)
Oropharyngeal cancer	63 (11.7)
Anal cancer	97 (18)
Genital warts	69 (12.8)
I don't know	279 (51.8)
Which one do you think are ways of preventing HPV diseases?*	
Practicing abstinence	59 (10.9)
Vaccination	206 (38.1)
Using condoms	183 (33.9)
It cannot be prevented	2 (0.4)
I don't know	276 (51.1)
What are the risk factors for HPV infection?*	
High frequency of sex partner exchange	188 (34.8)
Genital skin-to-skin contact	69 (12.8)
Body fluids (blood)	105 (19.4)
I don't know	306 (56.7)

Out of all the participants, nearly one-third had heard about the HPV vaccine. A major chunk was still not aware of this. Out of 160, who had heard about the vaccine, almost two-thirds of them agreed that both men and women should get this vaccine. More than 90% of voters believe that the HPV vaccine prevents cervical cancer. Vaginal cancer was seen as preventable by nearly half of the respondents. When asked about the recommended dose and ideal time for the HPV vaccine, 75% of students said two doses, and 83.8% said 9-14 years, respectively (Table 3).

**Table 3:** Distribution of study participants according to knowledge of HPV vaccination *Multiple responses HPV: human papillomavirus

Variables	Frequency (%)
Heard of HPV vaccine uses to prevent HPV infection (n=540)	
Yes	160 (29.6)
No	380 (70.4)
Who should get an HPV vaccination? (n=160)	
Only men	0 (0)
Only women	51 (31.9)
Men and women	105 (65.6)
I don't know	4 (2.5)
The HPV vaccine helps to:*	
Prevent cervical cancer	153 (95.6)
Prevent vaginal cancer	73 (45.6)
Prevent vulval cancer	17 (10.6)
I don't know	4 (2.5)
Recommended doses of the HPV vaccine	
1	13 (8.1)
2	120 (75)
I don't know	27 (16.9)
The ideal time HPV vaccine is best recommended.	
<9 years	5 (3.1)
9-14 years	134 (83.8)
Can be provided at any age	13 (8.1)
I don't know	8 (5)

Among 540 participants, only 3.3% (18) had taken the HPV vaccine. Out of them, 10 students had taken three doses, and eight students had taken only two doses. Out of these, a majority had prior information about the same. Health workers encouraged more than two-fifths of the participants, while their parents influenced one-third. Out of 522 participants who didn't receive the vaccine, the topmost reason was that they didn't have information about the vaccine (86.2%), followed by fear of needles (9.8%) (Table 4).

**Table 4:** Distribution of study participants according to uptake of HPV vaccine (n=540) *Multiple responses HPV: human papillomavirus

Questions	Frequency (%)
Have you received an HPV vaccination?	
Yes	18 (3.3)
No	522 (96.7)
If yes, how many doses of the vaccine?	
1	0 (0)
2	8 (44.4)
3	10 (55.6)
If you received it, what helped you receive it?*	
Prior information	12 (70.6)
Believes in its benefits	2 (11.8)
Encouragement from a health worker	7 (41.2)
Parental influence	6 (35.3)
Peer influence	1 (5.9)
Because it is cost-free	1 (5.9)
If you haven't taken the HPV vaccine before, why?*	
I have no information about the vaccine	451 (86.2)
I have a negative attitude toward the vaccine	11 (2.1)
Being absent on vaccination day	9 (1.7)
Fear of side effects	16 (3.1)
Fear of needle injection	51 (9.8)
Parental concern	22 (4.2)
Peer influence	8 (1.5)
Less belief in its benefit	16 (3.1)
Access	43 (8.2)
Societal pressures/rumors	12 (2.3)

Table 5 shows that more than 80% had inadequate knowledge regarding cervical cancer and HPV infection, but nearly 60% of participants had adequate knowledge about HPV vaccination.

**Table 5:** Distribution of study participants according to adequate and inadequate knowledge HPV: human papillomavirus

Variables	Frequency (%)
Knowledge of cervical cancer (n=265)	
Adequate knowledge	45 (16.98)
Inadequate knowledge	220 (83.02)
Knowledge of HPV infection (n=540)	
Adequate knowledge	88 (16.3)
Inadequate knowledge	452 (83.7)
Knowledge of HPV vaccination (n=160)	
Adequate knowledge	95 (59.4)
Inadequate knowledge	65 (40.6)

From this table, we can see that students with the following demographic profiles had adequate knowledge of cervical cancer: nearly one-third of the 16-18-year age group and studying in the 12th standard, one-third who came from rural residence and living with family, those who are living in joint family (38.7%), and more than two-fifths who had single parent and who had ≥2 siblings. Although many had adequate knowledge, significance was found only among those who had ≥2 siblings (Table 6).

**Table 6:** Association of the sociodemographic characteristics of the study participants with knowledge of cervical cancer in n (%) The chi-squared test was used, and a p-value of <0.05 was considered statistically significant

Variables	Adequate knowledge (n=90)	Inadequate knowledge (n=66)	Test of significance
Total	90 (34)	175 (66)	-
Age group (years)			
14-16	20 (31.3)	44 (68.8)	χ²=1.822; p=0.402
16-18	63 (33.7)	124 (66.3)	
≥18	7 (50)	7 (50)	
Class			
11th	32 (30.2)	74 (69.8)	χ²=1.122; p=0.290
12th	58 (36.5)	101 (63.5)	
Residence			
Urban	58 (32.6)	120 (67.4)	χ²=0.459; p=0.498
Rural	32 (36.8)	55 (63.2)	
Living status for the last 1 year			
With family	89 (34)	173 (66)	χ²=0.001; p=0.982
Living alone	1 (33.3)	2 (66.7)	
Religion			
Hindu	88 (34.1)	170 (65.9)	χ²=1.112; p=0.774
Muslim	2 (40)	3 (60)	
Sikh	0 (0)	1 (100)	
Other	0 (0)	1 (100)	
Type of family			
Joint	43 (38.7)	68 (61.3)	χ²=1.943; p=0.163
Nuclear	47 (30.5)	107 (69.5)	
Parents' marital status			
Both parents	83 (33.5)	165 (66.5)	χ²=0.422; p=0.516
Single parent	7 (41.2)	10 (58.8)	
Number of siblings			
None	4 (26.7)	11 (73.3)	χ²=7.620; p=0.022
<2	45 (28.3)	114 (71.7)	
≥2	41 (45.1)	50 (54.9)	

This table shows the association of participants' knowledge of cervical cancer with the education and occupation of their parents. The following students showed adequate knowledge: students whose only father was earning and whose father and mother had secondary education (37.2% and 41.6%, respectively) and one-third of whose fathers work in the government sector and whose mothers are housewives (35.6%). Although many had adequate knowledge, significance was found only among those who had ≥2 siblings (Table 7).

**Table 7:** Association of parents' education and occupation of the study participants with knowledge of cervical cancer in n (%) The chi-squared test was used, and a p-value of <0.05 was considered statistically significant

Variables	Adequate knowledge (n=90)	Inadequate knowledge (n=66)	Test of significance
Total	90 (34)	175 (66)	-
Employment status of parents			
Both unemployed	13 (40.6)	19 (59.4)	χ²=2.611; p=0.456
Both employed	16 (26.2)	45 (73.8)	
Only the father earns	58 (35.2)	107 (64.8)	
Only the mother earns	3 (42.9)	4 (57.1)	
Father's education			
Unable to read and write	2 (33.3)	4 (66.7)	χ²=0.601; p=0.963
Able to read and write	12 (32.4)	25 (67.6)	
Primary education	10 (32.3)	21 (67.7)	
Secondary education	32 (37.2)	54 (62.8)	
College diploma and above	34 (32.4)	71 (67.6)	
Mother's education			
Unable to read and write	5 (41.7)	7 (58.3)	χ²=3.592; p=0.464
Able to read and write	10 (31.3)	22 (68.8)	
Primary education	14 (31.8)	30 (68.2)	
Secondary education	32 (41.6)	45 (58.4)	
College diploma and above	29 (29)	71 (71)	
Father's occupation			
Govt. employee	28 (35)	52 (65)	χ²=0.670; p=0.715
Private employee	43 (31.9)	92 (68.1)	
Farmer	19 (38)	31 (62)	
Mother's occupation			
Govt. employee	8 (25)	24 (75)	χ²=1.741; p=0.628
Private employee	9 (31)	20 (69)	
Farmer	1 (50)	1 (50)	
Housewife	72 (35.6)	130 (64.4)	

According to the table above, students who met the following demographic categories knew enough about HPV infection: less than 25% of adolescents in every age group knew more than 12th graders (9.1%), students who lived with their families, students who had both parents (16.7%), and students who had more than two siblings (18.8%). Although many had adequate knowledge, no significance was found among the sociodemographic characteristics of study participants with respect to knowledge of HPV infection (Table 8).

**Table 8:** Association of the sociodemographic characteristics of the study participants with knowledge of HPV infection in n (%) The chi-squared test was used, and a p-value of <0.05 was considered statistically significant

Variables	Adequate knowledge (n=88)	Inadequate knowledge (n=452)	Test of significance
Total	88 (16.3)	452 (83.7)	-
Age group (years)			
14-16	18 (14.4)	107 (85.6)	χ²=1.066; p=0.587
16-18	63 (16.4)	320 (83.6)	
≥18	7 (21.9)	25 (78.1)	
Class			
11th	23 (9.1)	231 (90.9)	χ²=18.435; p=0.001
12th	65 (22.7)	221 (77.3)	
Residence			
Urban	59 (17.3)	283 (82.7)	χ²=0.624; p=0.430
Rural	29 (14.6)	169 (85.4)	
Living status for the last 1 year			
With family	85 (16)	445 (84)	χ²=1.525; p=0.467
With relatives	1 (25)	3 (75)	
Living alone	2 (33.3)	4 (66.7)	
Religion			
Hindu	86 (16.2)	445 (83.8)	χ²=1.167; p=0.761
Muslim	2 (28.6)	5 (71.4)	
Sikh	0 (0)	1 (100)	
Other	0 (0)	1 (100)	
Type of family			
Joint	37 (16.6)	186 (83.4)	χ²=0.024; p=0.876
Nuclear	51 (16.1)	266 (83.9)	
Parents' marital status			
Both parents	85 (16.7)	423 (83.3)	χ²=1.195; p=0.274
Single parent	3 (9.4)	29 (90.6)	
Number of siblings			
None	7 (28)	18 (72)	χ²=5.111; p=0.078
<2	41 (13.6)	261 (86.4)	
≥2	40 (18.8)	173 (81.2)	

This table shows the association of participants' knowledge of HPV infection with the education and occupation of their parents. The following students showed adequate knowledge: whose father was unable to read and write (27.3%), whose mother had done a secondary education (20.3%), whose father was a government employee (17%), and whose mother was a private employee (17.2%) (Table 9).

**Table 9:** Association of parents' education and occupation of the study participants with knowledge of HPV infection in n (%) The chi-squared test was used, and a p-value of <0.05 was considered statistically significant

Variables	Adequate knowledge (n=88)	Inadequate knowledge (n=452)	Test of significance
Total	88 (16.3)	452 (83.7)	-
Employment status of parents			
Both unemployed	11 (16.7)	55 (83.3)	χ²=0.486; p=0.922
Both employed	19 (16.7)	95 (83.3)	
Only the father earns	56 (16.4)	285 (83.6)	
Only the mother earns	2 (10.5)	17 (89.5)	
Father's education			
Unable to read and write	3 (27.3)	8 (72.7)	χ²=6.443; p=0.168
Able to read and write	11 (12.5)	77 (87.5)	
Primary education	9 (10.8)	74 (89.2)	
Secondary education	24 (15.2)	134 (84.8)	
College diploma and above	41 (20.5)	159 (79.5)	
Mother's education			
Unable to read and write	6 (19.4)	25 (80.6)	χ²=3.749; p=0.441
Able to read and write	10 (11.2)	79 (88.8)	
Primary education	14 (16.5)	71 (83.5)	
Secondary education	29 (20.3)	114 (79.7)	
College diploma and above	29 (15.1)	163 (84.9)	
Father's occupation			
Govt. employee	26 (17)	127 (83)	χ²=0.076; p=0.963
Private employee	43 (16)	225 (84)	
Farmer	19 (16)	100 (84)	
Mother's occupation			
Govt. employee	8 (13.6)	51 (86.4)	χ²=0.784; p=0.853
Private employee	11 (17.2)	53 (82.8)	
Farmer	0 (0)	2 (100)	
Housewife	69 (16.6)	346 (83.4)	

From the above table, we can see that students with the following demographic profiles had adequate knowledge of HPV vaccination: nearly two-thirds of the 14-16-year age group, three-fifths of students residing in urban area, more than half of participants living with family and belonging to the Hindu religion and living in joint family, and three-fifths of those who had both parents and had <2 siblings with only father was earning (Table 10).

**Table 10:** Association of the sociodemographic characteristics of the study participants with knowledge of HPV vaccination in n (%) The chi-squared test was used, and a p-value of <0.05 was considered statistically significant *Fisher's exact test was used, and a p-value of <0.05 was considered statistically significant

Variables	Adequate knowledge (n=95)	Inadequate knowledge (n=65)	Test of significance
Total	95 (59.4)	65 (40.6)	-
Age group (years)			
14-16	22 (68.8)	10 (31.3)	χ²=2.074; p=0.354
16-18	70 (57.9)	51 (42.1)	
≥18	3 (42.9)	4 (57.1)	
Class			
11th	35 (62.5)	21 (37.5)	χ²=0.349; p=0.555
12th	60 (57.7)	44 (42.3)	
Residence			
Urban	73 (60.3)	48 (39.7)	χ²=0.188; p=0.665
Rural	22 (56.4)	17 (43.6)	
Living status for the last 1 year			
With family	93 (58.9)	65 (41.1)	p=0.515*
Living alone	2 (100)	0 (0)	
Religion			
Hindu	93 (58.9)	65 (41.1)	p=0.239*
Muslim	2 (100)	0 (0)	
Type of family			
Nuclear	32 (51.6)	30 (48.4)	χ²=2.528; p=0.112
Joint	63 (64.3)	35 (35.7)	
Parents' marital status			
Both parents	89 (61)	57 (39)	χ²=1.735; p=0.188
Single parent	6 (42.9)	8 (57.1)	
Number of siblings			
None	4 (40)	6 (60)	χ²=1.676; p=0.433
<2	58 (61.1)	37 (38.9)	
≥2	33 (60)	22 (40)	

This table shows the association of participants' knowledge of HPV vaccination with the education and occupation of their parents. The following students showed adequate knowledge: whose father and mother had education above college and diploma, more than half of the students whose fathers work in the private sector, and three-fifths of whose mothers were housewives (Table 11).

**Table 11:** Association of parents' education and occupation of the study participants with knowledge of HPV vaccination in n (%) The chi-squared test was used, and a p-value of <0.05 was considered statistically significant

Variables	Adequate knowledge (n=95)	Inadequate knowledge (n=65)	Test of significance
Total	95 (59.4)	65 (40.6)	-
Employment status of parents			
Both unemployed	9 (60)	6 (40)	χ²=1.840; p=0.606
Both employed	24 (58.5)	17 (41.5)	
Only the father earns	60 (61.2)	38 (38.8)	
Only the mother earns	2 (33.3)	4 (66.7)	
Father's education			
Unable to read and write	1 (25)	3 (75)	χ²=2.206; p=0.698
Able to read and write	14 (58.3)	10 (41.7)	
Primary education	12 (57.1)	9 (42.9)	
Secondary education	28 (62.2)	17 (37.8)	
College diploma and above	40 (60.6)	26 (39.4)	
Mother's education			
Unable to read and write	6 (54.5)	5 (45.5)	χ²=1.738; p=0.784
Able to read and write	13 (68.4)	6 (31.6)	
Primary education	13 (52)	12 (48)	
Secondary education	31 (63.3)	18 (36.7)	
College diploma and above	32 (57.1)	24 (42.9)	
Father's occupation			
Govt. employee	28 (60.9)	18 (39.1)	χ²=0.062; p=0.969
Private employee	53 (58.9)	37 (41.1)	
Farmer	14 (58.3)	10 (41.7)	
Mother's occupation			
Govt. employee	10 (52.6)	9 (47.4)	χ²=2.022; p=0.568
Private employee	12 (54.5)	10 (45.5)	
Farmer	2 (100)	0 (0)	
Housewife	71 (60.7)	46 (39.3)	

Table 12 shows the binary logistic regression analysis and reveals that the odds of having adequate knowledge of HPV infection were 2.968 times significantly higher (AOR=2.968; 95% CI=1.752-5.030) among participants from the 12th standard. The odds of having adequate knowledge of HPV infection were 0.297 times lower (AOR=0.297; 95% CI=0.113-0.784) among participants who had less than two siblings. The odds of taking the HPV vaccine were 0.196 times significantly lower (AOR=0.196; 95% CI=0.073-0.528) among participants having adequate knowledge of HPV infection.

**Table 12:** Factors associated with knowledge of cervical cancer, HPV infection, and HPV vaccine Estimated by logistic regression analysis HPV: human papillomavirus

Variables		Crude odds ratio (95% CI)	Adjusted odds (95% CI)
Knowledge of cervical cancer (n=265)	Type of family		
	Joint	1	1
	Nuclear	0.695 (0.416-1.161)	0.677 (0.402-1.141)
	Number of siblings		
	None	1	1
	<2	1.086 (0.329-3.587)	1.106 (0.333-3.671)
	≥2	2.255 (0.668-7.612)	2.325 (0.685-7.894)
Knowledge of HPV infection (n=540)	Class		
	11th	1	1
	12th	2.954 (1.774-4.919)	2.968 (1.752-5.030)
	Number of siblings		
	None	1	1
	<2	0.404 (0.159-1.027)	0.297 (0.113-0.784)
	≥2	0.595 (0.233-1.519)	0.470 (0.178-1.245)
	Father's education		
	Unable to read and write	1	1
	Able to read and write	0.381 (0.088-1.656)	0.372 (0.083-1.674)
	Primary education	0.324 (0.073-1.448)	0.299 (0.064-1.391)
	Secondary education	0.478 (0.118-1.929)	0.502 (0.120-2.103)
	College diploma and above	0.688 (0.175-2.708)	0.717 (0.176-2.925)
	HPV vaccine uptake		
	Present	1	1
	Absent	0.178 (0.069-0.463)	0.196 (0.073-0.528)
Knowledge of HPV vaccine (n=160)	Type of family		
	Joint	1	1
	Nuclear	1.687 (0.883-3.223)	1.648 (0.859-3.160)
	Parents' marital status		
	Both parents	1	1
	Single parent	0.480 (0.158-1.457)	0.506 (0.165-1.548)

## Discussion

Our study found a significant gap in awareness regarding cervical cancer and its prevention among adolescents, because less than half were aware of cervical cancer and the HPV infection and less than one-third had heard about the HPV vaccine. Comparatively, a study found that a significantly higher proportion of girls were aware of cervical cancer. Still, less than one-third of the participants had ever heard of HPV infection [12]. This indicates that while general awareness of cervical cancer was high, knowledge of its specific causative agent, HPV, was relatively low. Regarding knowledge about cervical cancer, the majority had inadequate knowledge; only one-fifth of the participants had adequate knowledge. This coincides with studies done by Abiodun et al. in Nigeria [13]. Likewise, we found that nearly three-fifths of the participants had adequate knowledge about the HPV vaccine. Still, a large portion of the participants were unaware of the vaccine. This highlights the need for targeted education to increase awareness and knowledge about the HPV vaccine. Another study done by Lakneh et al. in Ethiopia supports our result [14]. In our study, only a small proportion of participants had received the HPV vaccine, with 18 out of 540 individuals reporting vaccination. On the other hand, the majority of participants had not received the vaccine, with the most common reason being a lack of information, followed by fear of needle injections. A study done by Beyen et al. in Ethiopia reported that nearly half of the participants had received at least one dose of the HPV vaccine and the remaining half had reasons like lack of pre-information, fear of side effects, and being absent during vaccination days [15].

When it comes to sociodemographic association with knowledge of cervical cancer and HPV infection, adequate knowledge was highest among participants aged ≥18 years. This suggests that older participants may have better access to health education and exposure to related topics in schools or through social networks. A study done by Turhan et al. in Turkey also supports our result, which found that older adolescents (≥18 years) had greater knowledge about HPV infection and cervical cancer, attributing this to age-related exposure to sex education and healthcare interactions [16]. In our study, the participants with the following sociodemographic characteristics had exhibited adequate knowledge of cervical cancer HPV infection: those who are in the 12th grade and from urban residents, those who are living in a joint family, and those who had ≥2 siblings, which may indicate that larger families engage more in health-related discussions; whose fathers and mothers had secondary education; and whose fathers were farmer and mothers were housewife which suggested that housewives often spend more time engaging with their children, contributing to better knowledge transmission. Though these characteristics show adequate knowledge, only those with ≥2 siblings were found to be significant with knowledge of cervical cancer, and those who are in the 12th grade were significant with knowledge of the HPV vaccine. A similar result was found in a study done by Brohman et al., that is, that peers and siblings were the source of vaccine information for students [17]. Another study done by Rashid et al. found that female students who are in higher grades have better knowledge about the HPV vaccine than their younger counterparts [18].

In contrast to the findings with knowledge of cervical cancer and HPV infection, the participants with the following sociodemographic characteristics exhibited adequate knowledge of HPV vaccination: younger adolescents (14-16 years), those who were in the 11th grade and from urban area, those who were living with both parents in a joint family, those who had <2 siblings, and those whose fathers and mothers had secondary education. Though these characteristics show adequate knowledge, no statistical significance was found among them.

In our study, binary logistic regression analysis showed that participants in the 12th standard had significantly higher odds of having adequate knowledge about HPV infection. Those who had fewer than two siblings had significantly lower adequate knowledge of HPV infection. Additionally, participants with inadequate knowledge of HPV infection had significantly lower odds of taking the HPV vaccine (AOR=0.196). These findings align with the study by Mihretie et al. in Debre Tabor Town, Ethiopia, which found that adolescents with access to information and those aged 13-15 years were more likely to have good knowledge of the HPV vaccine. The study also emphasized that having a source of information significantly increased the likelihood of good knowledge [19].

In this study, awareness and knowledge regarding cervical cancer, HPV infection, and the HPV vaccine among female adolescents were found to be alarmingly low. Only half of the participants were aware of cervical cancer, with a significant number of those having inadequate knowledge. Regarding HPV infection, only 16.3% of the participants had adequate knowledge, and 83.7% lacked awareness. Furthermore, awareness of the HPV vaccine was even lower, and among those who are aware of the vaccine, only three-fifths had adequate knowledge about it. The study also highlighted the significant impact of education level and family background on knowledge and vaccine uptake. Participants in the 12th grade, those with fathers having secondary education, and those with adequate knowledge of HPV infection were more likely to take the vaccine.

Strengths

This study has some strengths. To the best of our knowledge, this is the first study conducted in the Sonipat district on this topic in the past five years. The sample size was adequate, and it was calculated with α at a 95% confidence interval. The data was collected by a single investigator only, which helps in avoiding inter-observer bias in the study. A focus group discussion was conducted to get a glimpse of the awareness and knowledge among teachers who were teaching the study participants.

Limitations

This study has some limitations. The generalizability of this study is limited to the district only. This study employed a cross-sectional design, which limits the ability to interpret the direction of causality. The study participants may have responded in such a way as to portray themselves in a good light, so the probability of social desirability bias cannot be ruled out. Being a cross-sectional study, recall bias could remain a possibility. It could have involved parents' participation, too, in the focus group discussion.

## Conclusions

The research shows a significantly positive correlation between increased knowledge (even basic knowledge) and a high willingness or intention to receive the vaccine in the future, given accessibility and affordability. Despite the low uptake, the study indicates a foundational level of general awareness about the HPV vaccine, but there is also a critical need for comprehensive awareness campaigns and educational interventions targeting HPV and cervical cancer prevention. Further studies are needed to explore the factors influencing vaccine uptake and to evaluate the effectiveness of targeted educational programs to increase vaccine coverage among adolescents.

## Appendices

Appendix A

**Table 13:** Sociodemographic characteristics of the participants Questionnaire created by the authors

Participants' sociodemographic characteristics	
Age (in years)	
Class	
Residence	Rural/Urban
Religion	Hindu/Muslim/Sikh/Other
Family type	Nuclear/Joint
Parents' marital status	Non-single parent/Single parent
No. of siblings	None/>2/<2
Job of parents	Both unemployed/Both employed/Only the father earns/Only the mother earns
Living status (from the last 1 year)	With family/Living alone
Father's education	Unable to read and write/Able to read and write/Primary education/Secondary education/College or diploma and above
Mother's education	Unable to read and write/Able to read and write/Primary education/Secondary education/College or diploma and above
Father's occupation	Govt. employee/Private employee/Farmer
Mother's occupation	Govt. employee/Private employee/Farmer/Housewife

Appendix B

**Table 14:** Knowledge of the study participants about cervical cancer, HPV infection, HPV vaccination, and HPV vaccine uptake HPV: human papillomavirus Questionnaire created by the authors

Section A: Knowledge of the study participants about cervical cancer		
A.1	Have you ever heard of cervical cancer?	Yes/No (If No, skip to Q-B.1)
A.2	Cervical cancer is a common female cancer	Yes/No/I don't know
A.3	Organs affected by cervical cancer	Uterus/Breast/I don't know/Others (specify)
A.4	Mode of acquisition of cervical cancer	Sexual transmission/History of cancer in a family/I don't know/Others (specify)
A.5	What do you think is/are the risk factor(s) for cervical cancer? (more than one answer possible)	HPV infection/Promiscuity/Early age onset of sexual activity/Smoking/History of sexually transmitted infection/I don't know
A.6	When do you recognize the signs and symptoms of cervical cancer? When there is:	Abnormal vaginal bleeding/Vaginal discharge/Dyspareunia/I don't know
Section B: Knowledge towards HPV infection		
B.1	HPV is a virus that causes sexually transmitted infections. Have you ever heard of it?	Yes/No
B.2	Who can contract HPV infection?	Only men/Only women/Men and women/I don't know
B.3	Which of these diseases are caused by HPV infection? (more than one answer possible)	Cervical cancer/Penile cancer/Oropharyngeal cancer/Anal cancer/Genital warts/I don't know
B.4	Which one do you think are ways of preventing HPV diseases? (more than one answer possible)	Practicing abstinence/Vaccination/Using condoms/Cannot be prevented/I don't know
B.5	What are the risk factors for HPV infection? (more than one answer possible)	High frequency of sex partner exchange/Genital skin-to-skin contact/Body fluids (blood)/I don't know
Section C: Knowledge towards HPV vaccination		
C.1	The HPV vaccine is a vaccine used to prevent HPV infection. Have you ever heard of it?	Yes, I have/No, I haven't (If No, skip to Q-D.1)
C.2	Who should get the HPV vaccination?	Only men/Only women/Men and women/I don't know
C.3	The HPV vaccine helps to:	Prevent cervical cancer/Prevent vaginal cancer/Prevent vulva cancer/I don't know/Others (specify)
C.4	Recommended doses of the HPV vaccine	One dose/Two doses/I don't know
C.5	The ideal time HPV vaccine is best recommended	<9 years/9-14 years/Can be provided at any age/I don't know
Section D: HPV vaccine uptake		
D.1	Have you received an HPV vaccination?	Yes, I have/No, I haven't (If No, skip to Q-D.4)
D.2	If Yes, how many doses of the vaccine?	One/Two/Three
D.3	If you received, what helped you to receive? (possibly more than one answer)	Prior information/Believes in its benefit/Encouragement from a health worker/Parental influence/Peer influence/Because it is cost-free/Others (specify)
D.4	If you haven't taken the HPV vaccine before, why? (skip if you have received)	I have no information about the vaccine/I have a negative attitude toward the vaccine/Being absent on vaccination day/Fear of side effects/Fear of needle injection/Parental concern/Peer influence/Less belief in its benefit/Access/Social pressures/rumors/Others (specify)
